# Supervised Home-Based Exercise Intervention in Colorectal Cancer Patients Following Surgery: A Feasibility Study

**DOI:** 10.3390/ijerph22040524

**Published:** 2025-03-30

**Authors:** Eleonora Latini, Attilio Parisi, Claudia Cerulli, Elisa Grazioli, Eliana Tranchita, Arianna Murri, Paolo Mercantini, Alessio Lucarini, Marcello Gasparrini, Lorenzo Ridola, Luca Tagliente, Flavia Santoboni, Donatella Trischitta, Mario Vetrano, Vincenzo Visco, Maria Chiara Vulpiani, Sveva Maria Nusca

**Affiliations:** 1 Physical Medicine and Rehabilitation Unit, Department of Medical and Surgical Sciences and Translational Medicine, Sant’Andrea University Hospital, “Sapienza” University of Rome, 00189 Rome, Italy; luca.tagliente@uniroma1.it (L.T.); flavia.santoboni@ospedalesantandrea.it (F.S.); dtrischitta@ospedalesantandrea.it (D.T.); mario.vetrano@uniroma1.it (M.V.); mariachiara.vulpiani@uniroma1.it (M.C.V.); sveva.nusca@uniroma1.it (S.M.N.); 2 Department of Movement, Human and Health Sciences, University of Rome “Foro Italico”, 00135 Rome, Italy; attilio.parisi@uniroma4.it (A.P.); claudia.cerulli@uniroma4.it (C.C.); elisa.grazioli@uniroma4.it (E.G.); eliana.tranchita@gmail.com (E.T.); arianna.murri@uniroma4.it (A.M.); 3 Department of Medical and Surgical Sciences and Translational Medicine, Sant’Andrea University Hospital,“Sapienza” University of Rome, 00189 Rome, Italy; paolo.mercantini@uniroma1.it (P.M.); alessio.lucarini@uniroma1.it (A.L.); 4 Department of General Surgery, Sant’Andrea University Hospital, “Sapienza” University of Rome, 00189 Rome, Italy; marcello.gasparrini@ospedalesantandrea.it; 5 Department of Medical and Surgical Sciences and Biotechnologies, “Sapienza” University of Rome, 00185 Rome, Italy; lorenzo.ridola@uniroma1.it; 6 Department of Clinical and Molecular Medicine, Sant’Andrea University Hospital, “Sapienza” University of Rome, 00189 Rome, Italy; vincenzo.visco1@uniroma1.it

**Keywords:** post-operative rehabilitation, colorectal surgery, oncological rehabilitation, physical exercise, combined exercise, supervised exercise, Enhanced Recovery After Surgery (ERAS), quality of life

## Abstract

This pilot study aimed to assess the feasibility and preliminary effects of a supervised, home-based exercise program in patients recovering from laparoscopic colorectal cancer surgery. A total of 23 patients were included, with 13 participating in the exercise intervention and 10 receiving standard postoperative care. The exercise group (intervention group) followed a two-month structured program, while the control group received no structured exercise. Feasibility was demonstrated by 98% adherence in the intervention group and no reported adverse events. At T1, the intervention group showed significant improvements in role function, cognitive function, and reduced fatigue compared to the control group. At T2, a significant difference was observed in physical function. Functional capacity, assessed by the Six-Minute Walk Test, was significantly better in the intervention group at T1, T2, and T3, as was physical performance measured by the Short Physical Performance Battery (SPPB) at T1, T2, and T3. No significant differences were observed between the groups in anxiety, depression, sleep quality, or body composition parameters. This study highlights the feasibility of a supervised home-based exercise program in the early postoperative phase, demonstrating positive effects on Quality of Life, functional recovery, and fatigue in colorectal cancer patients.

## 1. Introduction

Colorectal cancer is one of the most common malignancies globally and remains a significant public health challenge [1]. In 2023, the prevalence of cancer in Italy exceeded 3.7 million cases, with colorectal cancer accounting for 50,500 new diagnoses annually [2]. It is the third most frequent cancer in men and the second in women. Advances in screening programs and modern treatments have improved five-year survival rates to approximately 65%, yet survivors often face substantial long-term complications [2].

Postoperative recovery in colorectal cancer patients is frequently characterized by fatigue, reduced physical performance, and impaired quality of life (QoL). These limitations, compounded by physical deconditioning, changes in body composition, and psychological distress, highlight the critical need for effective rehabilitative strategies [3]. Antitumor treatments, such as chemotherapy and other adjuvant therapies, often exacerbate these challenges by inducing treatment-related fatigue, which can significantly affect both physical and psychological well-being. Recent evidence suggests that only a minority of survivors regain pre-diagnosis levels of functionality and QoL, underscoring the persistent impact of the disease and its treatments [4].

Rehabilitation interventions, particularly those incorporating physical exercise, have shown promise in mitigating the adverse effects of cancer therapies [5,6]. Exercise has demonstrated efficacy in improving cardiorespiratory fitness, muscle strength, and metabolic health, as well as reducing fatigue, anxiety, and depression [5,6]. Since physical functionality is strongly correlated with quality of life, enhancing functional capacity through structured exercise programs may be a key factor in optimizing recovery and long-term well-being in colorectal cancer survivors. Moreover, exercise may attenuate systemic inflammation—a key driver of cancer progression and treatment-related side effects—by modulating pro-inflammatory cytokines such as TNF-α, IL-1, and IL-6 [7,8,9,10,11]. These mechanisms may contribute to improved clinical outcomes and reduced recurrence rates in cancer survivors [7,8,9,10,11].

The Enhanced Recovery After Surgery (ERAS) protocol emphasizes early mobilization as a cornerstone of postoperative care, aiming to reduce perioperative stress and enhance recovery [12]. However, structured and supervised exercise programs specifically tailored to the needs of colorectal cancer patients are not yet part of standard postoperative clinical practice. Most studies exploring exercise interventions have focused on programs initiated weeks or months after surgery [13,14,15], leaving a gap in knowledge regarding the potential benefits of early postoperative exercise.

The current literature highlighted the positive effects of physical activity on fatigue, fitness, and QoL in colorectal cancer patients [13]. However, only a limited number of studies have examined the clinical impact of exercise programs initiated immediately after surgery [16,17]. Early postoperative exercise may offer unique benefits, as this period is critical for counteracting physical deconditioning and fostering functional recovery.

This study aimed to evaluate the feasibility and preliminary effects of a supervised, home-based exercise program initiated shortly after laparoscopic colorectal cancer surgery. By assessing its impact on QoL, functional capacity, and physical performance, this research seeks to fill a critical gap in the literature and provide evidence to support the integration of structured exercise into standard postoperative care. The findings may contribute to the development of targeted rehabilitation protocols that enhance recovery and long-term outcomes in colorectal cancer patients.

In addition, feasibility research, as conducted in this study, serves a crucial role in determining whether an intervention is worth further investigation. By evaluating factors such as recruitment rates, adherence to the protocol, and incidence of adverse events, this study helps establish the viability of integrating a home-based exercise program into routine clinical practice, thereby guiding future, larger-scale clinical trials. As highlighted in the literature, using feasibility studies to assess an intervention’s practicality is a key step in advancing only those interventions that demonstrate potential for meaningful clinical benefit.

### 1.1. The Primary Objective of the Study

The primary objective of this study was to evaluate the effects of a supervised, home-based physical exercise program lasting two months on QoL in a group of patients undergoing laparoscopic surgery for colorectal cancer, compared to those receiving standard care, at 2 months from the start of the exercise program (end of the rehabilitation treatment) (T1) and at 2 (T2) and 4 (T3) months after the end of the treatment.

### 1.2. Secondary Objectives of the Study

1. To evaluate the effects of physical exercise on patients’ functional capacity, physical performance, muscle strength, muscle mass, nutritional and hydration status, body composition, levels of anxiety and depression, and sleep quality in the intervention group (IG) compared to the control group (CG) at the different follow-up times (T1, T2, T3).
2. To assess the above-mentioned outcomes within the IG and CG at each follow-up time point (T1, T2, T3) compared to baseline.
3. To evaluate the incidence and types of postoperative complications within the IG and CG.
4. To assess the feasibility and safety of the physical exercise intervention within the context of the clinical study, including recruitment rates, adherence to the protocol, intervention tolerability, and incidence of adverse events.

## 2. Materials and Methods

### 2.1. Study Design

This research represents a pilot study, whose preliminary findings will be validated through a subsequent Randomized Controlled Trial (RCT), which has received approval from the Institutional Review Board of the “Sapienza” University of Rome (RS 5304/2019). The study adheres to good clinical practice standards and follows the ethical principles outlined in the Helsinki declaration [18].

### 2.2. Eligibility Criteria and Enrollment

The recruitment period extended from November 2022 to March 2024 and took place at the Emergency Surgery Unit, Week-Day Surgery Unit, and General Surgery Unit of Sant’Andrea Hospital, Rome, Italy. A physiatrist from the Physical Medicine and Rehabilitation Unit of the same hospital managed patient recruitment. Eligible participants were individuals newly diagnosed with colorectal cancer who had undergone laparoscopic resective surgery with curative intent.

The inclusion criteria encompassed the following conditions: an age range of 18 to 80 years; a histologically confirmed diagnosis of localized primary colon or rectal cancer (stages I–III) treated with laparoscopic resection surgery; a physically inactive lifestyle (defined as engaging in less than 150 min of physical activity per week); a Karnofsky Performance Status (KPS) score above 70; and the ability to walk at least 60 m. The Karnofsky Performance Status is a widely used and validated scale for assessing the functional status of the cancer patient. A KPS ≤ 70 indicates significant impairment in performing daily activities and self-care, along with an inability to work [19].

**Figure 1:**
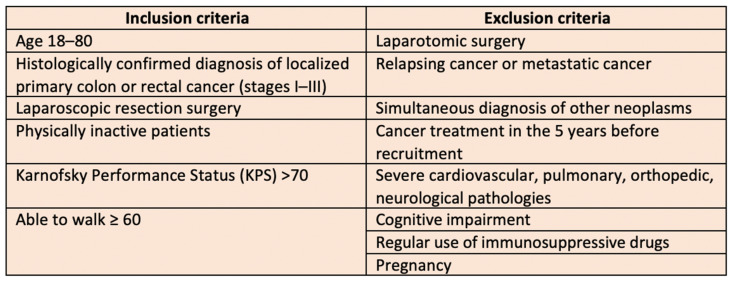
Inclusion and exclusion criteria.

Exclusion criteria included the following conditions: laparotomic surgery; pregnancy; recurrent or metastatic cancer; simultaneous diagnosis of additional malignancies; cancer treatment within the five years preceding recruitment; severe cardiovascular, pulmonary, orthopedic, neurological pathologies; cognitive impairment; regular use of immunosuppressive drugs. The inclusion and exclusion criteria are summarized in Figure 1.

**Figure 2:**
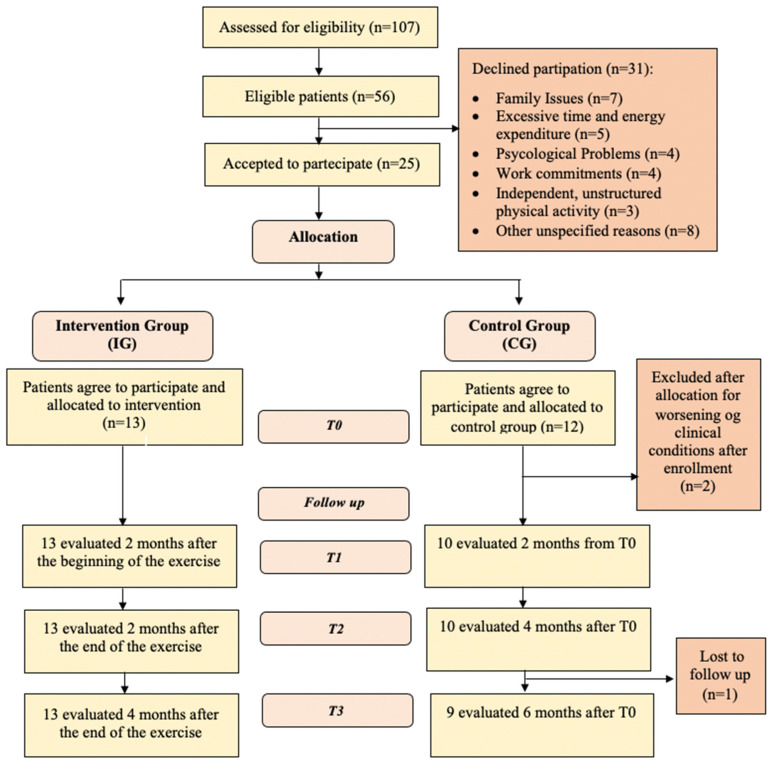
Consort flow diagram.

During the initial assessment, detailed explanations regarding the study were provided, and the inclusion and exclusion criteria were carefully evaluated. Patients who declined participation were asked to specify their reason, and their responses were documented. Eligible individuals who agreed to enroll provided written informed consent. A surgical evaluation was performed in conjunction with the first post-surgical follow-up visit, approximately ten days after the surgery, to determine suitability for physical activity. Subsequently, a cardiological screening was conducted by a Sports Medicine Specialist at “Foro Italico” University of Rome, Italy, to confirm eligibility for a physical exercise program. The participant flow throughout the study is illustrated in Figure 2. Patients included in the study were consecutively allocated to the IG (supervised home-based exercise program) and the CG (standard of care), following a non-randomized study design. Outcome measures were assessed before the start of the rehabilitation program (T0), at 2 months from the start of exercise (coinciding with the end of the treatment) (T1), and at 2 (T2) and 4 (T3) months after the end of the treatment. The assessments at T0, T1, T2, and T3 were conducted by the same specialist in Physical and Rehabilitation Medicine.

### 2.3. Intervention Group (Supervised and Home-Based Exercise)

Patients assigned to the IG participated in a two-month exercise program. The physical exercise was supervised and involved moderate-intensity aerobic exercise during the first month, followed by combined moderate-intensity aerobic and resistance exercises during the second month. Exercise sessions were conducted in the immediate postoperative phase, approximately 15–20 days after surgery, at the patients’ homes. A kinesiologist trained in the study protocol and a specialist in Physical and Rehabilitation Medicine supervised each session via telemedicine. The rehabilitation protocol was designed in accordance with the guidelines established by the American College of Sports Medicine (ACSM) [20] and the Canadian Society for Exercise Physiology (CSEP) [21], which recommend 150 min of moderate-intensity aerobic exercise per week alongside two to three weekly sessions of resistance training. Previous oncological research has demonstrated the safety and efficacy of both combined and aerobic exercise [22,23,24]. Each patient participated in three supervised exercise sessions per week, each lasting one hour. The structure of the session comprised a 10-min warm-up, 40 min of aerobic exercise during the first month post-surgery, followed by a combination of 20 min of aerobic activity and 20 min of muscle-strengthening exercises in the second month, and concluded with a 10-min cooldown phase. The sessions were to be discontinued in cases of specific adverse conditions: cardiovascular, pulmonary, or traumatic events; hemoglobin levels ≤ 10 g/dL (until restored above this threshold); neutropenia (absolute neutrophil count < 0.5 × 10^9^ μL); thrombocytopenia (platelet count < 50 × 10^9^ μL); sudden onset of nausea and vomiting within 24–36 h post-exercise; severe fatigue; decreased muscle strength; disorientation; vision disturbances; pain; newly peripheral neuropathies with reduced muscle strength; ataxia; and balance impairment. The rehabilitation regimen could be resumed once these conditions were resolved. Aerobic exercise was performed at a moderate intensity, maintaining 60–70% of the patient’s maximum heart rate, calculated using the Karvonen formula [25]. Adjustments to intensity were made according to subjective exertion levels assessed using the Borg Scale [26]. Each participant used a heart rate monitor to ensure adherence to prescribed intensity. Examples of home-based aerobic exercises include walking, step exercises, chair exercises, and other bodyweight exercises, all of which have been validated in the literature for improving cardiovascular fitness and quality of life in post-surgical colorectal cancer patients.

Strength training was conducted at 30–50% of the estimated one-repetition maximum (1-RM), determined using the Brzycki formula [27]. The 1-RM is a measure of maximal dynamic strength, indicating the heaviest weight a muscle or muscle group can lift in a single repetition with proper technique. The workload was progressively increased every two weeks when the patient rated the exercise as very light (Borg CR10 = 1). Two sets of ten repetitions were performed for each exercise, targeting the main muscle groups. Examples of home-based strengthening exercises include squats, lunges, glute bridges, and wall sits, all of which are effective for targeting major muscle groups and enhancing overall strength and function.

### 2.4. Control Group

Participants assigned to the CG were encouraged to maintain an active lifestyle. They received specific guidance on incorporating physical activity into their daily routines. For example, they were advised to choose stairs over elevators and to set aside time for daily walks to promote mobility and contribute to overall well-being. These activities were designed to encourage an active lifestyle without following a structured exercise program.

### 2.5. Outcome Measures

#### 2.5.1. Primary Outcome Measures

EORTC QLQ-C30: The quality of life was assessed using the Italian version of the EORTC QLQ-C30 (European Organization for Research and Treatment of Cancer Quality of Life-C30 questionnaire), a validated tool specifically designed for oncology patients. The questionnaire comprises nine multi-item scales: five functional scales (physical, role, cognitive, emotional, and social), three symptom scales (fatigue, pain, and nausea/vomiting), and one global health status scale. Additional single-item scales assess symptoms related to cancer or its treatment (dyspnea, insomnia, appetite loss, constipation, diarrhea) and the economic impact of the disease. Scoring interpretation follows the standard EORTC QLQ-C30 guidelines: higher scores on the global health scale indicate better quality of life, while higher scores on the functional scales indicate greater functional capacity, and higher symptom scores indicate worse symptom severity [28].

#### 2.5.2. Secondary Outcome Measures

1. Six-Minute Walk Test (6MWT): Functional capacity was measured through the 6MWT, a validated test that measures the maximum distance walked in six minutes, reflecting submaximal physical exercise capacity [29].
2. Short Physical Performance Battery (SPPB): Physical performance was evaluated using the SPPB [30].
3. Handgrip Strength Test: Muscular strength was measured using a portable dynamometer [31,32,33].
4. Skeletal Muscle Mass Index (SMI): Skeletal muscle mass was evaluated using bioelectrical impedance analysis (BIA), a non-invasive, rapid method recommended for clinical use [34,35,36].
5. Phase Angle (PhA): Nutritional status was assessed using the phase angle (PhA) derived from BIA parameters. PhA serves as an indicator of cellular health and integrity and is a prognostic marker in cancer patients [37].
6. Body composition: In addition to data on the Skeletal Muscle Index (SMI) and Phase Angle (PhA), body composition was assessed using BIA, analyzing parameters such as Resistance (Rz), Reactance (Xc), Fat-Free Mass (FFM), Fat Mass (FM), Total Body Water (TBW), Extracellular Water (ECW), Body Cell Mass (BCM), Skeletal Muscle Mass (SMM), Appendicular Skeletal Muscle Mass (ASMM), Fat Mass Percentage (FMpct), Fat-Free Mass Percentage (FFMpct), Fat Mass Index (FMI), Fat-Free Mass Index (FFMI), Hydration and Nutritional Status, along with additional parameters, providing a comprehensive overview of the patients’ health status.
7. Anxiety and Depression: The Hospital Anxiety and Depression Scale (HADS) was used to assess anxiety and depression levels [38].
8. Sleep Quality: was assessed using the Pittsburgh Sleep Quality Index (PSQI) [39,40].
9. Post-Operative Complications: Surgical complications were classified using the Dindo-Clavien classification, with grades ranging from I (bed rest) to IV (intensive care) [41].

## 3. Statistical Methods

Descriptive statistical analyses encompassed the median and range for continuous variables, while categorical variables were summarized using percentages and frequency tables. Given the limited sample size, a nonparametric statistical approach was adopted. To compare the IG and CG at the four time points (T0, T1, T2, T3), the Mann–Whitney U test was applied. Changes within each group across all follow-up assessments were evaluated using the nonparametric Wilcoxon signed-rank test. The analysis was conducted following the intention-to-treat principle. All statistical tests were two-tailed, with a significance threshold set at *p* < 0.05. Data processing and analysis were carried out using IBM SPSS Statistics version 20.0 (Chicago, IL, USA).

## 4. Results

Between November 2022 and March 2024, 107 patients with colorectal cancer were evaluated at the Emergency Surgery Unit, the Week-Day Surgery Unit, and the General Surgery Unit of the Sant’Andrea University Hospital in Rome. A total of 56 patients were deemed eligible for the study. Of these, 25 agreed to participate, while 31 declined. The reported reasons for refusal included 7 patients due to family reasons, 5 patients who believed the program required excessive time and effort, 4 patients due to psychological concerns, 4 patients because of work commitments, 3 patients who preferred to engage in unstructured physical activity independently, and 8 patients due to other unspecified reasons (Figure 2).

A total of 25 patients were assigned into two groups: 13 in the IG and 12 in the CG. However, two patients from the CG were excluded after allocation due to worsening physical conditions, resulting in a final population of 13 patients in the IG and 10 in the CG, for a total of 23 patients. In the IG, all 13 patients were evaluated at T1, T2, and T3, with no dropouts. In the CG, 10 patients were evaluated at T1 and T2, while at T3, 9 patients were assessed as one patient missed the final follow-up. The Consort Flow Diagram is presented in Figure 2. In the IG, 61.5% were male and 38.5% were female, while in the CG, 60% were male and 40% were female. The median age in the IG was 65.0 years (range: 56.0–80.0), while in the CG, it was 74.5 years (range: 71.0–78.0). The median BMI in the IG was 22.7 (range: 20.1–25.7), while in the CG it was 26.25 (range: 24.2–28.3). During the study period, a significant percentage of patients received adjuvant chemotherapy. Specifically, 9 patients (69.2%) in the IG and 5 patients (50%) in the CG initiated chemotherapy postoperatively.

No statistically significant differences (*p* > 0.05) were found between the groups at baseline. The demographic and clinical characteristics of the patients at baseline are summarized in Table 1.

### 4.1. Primary Outcome—EORTC QLQ-C30

Regarding the primary outcome, measured using the EORTC QLQ-C30, no statistically significant differences were observed between the IG and the CG at T0, indicating that the two groups were homogeneous at the beginning of the study (Table 2).

The between-group analysis revealed statistically significant differences at T1 between the IG and CG in favor of the IG for the following items: role function (*p* = 0.007), cognitive function (*p* = 0.013), and fatigue (*p* = 0.004). At T2, a statistically significant difference was observed in physical function (*p* = 0.006), also favoring the IG (Table 2).

The within-group analysis for the IG showed significant variations in the evaluated parameters at the different follow-up time points (T1, T2, and T3) compared to baseline (T0). Among the functional items, global quality of life and role function showed significant improvements across all time points compared to baseline, with *p*-values of 0.014 and 0.007 at T1, 0.018 and 0.043 at T2, and 0.014 and 0.012 at T3, respectively. Physical function also showed significant improvements at T1 (*p* = 0.021) and T2 (*p* = 0.018) compared to baseline; emotional function improved at T1 (*p* = 0.041) and T3 (*p* = 0.046); and social function improved at T2 (*p* = 0.026) and T3 (*p* = 0.011). Regarding symptom items, pain demonstrated significant improvement at T1 (*p* = 0.030) and T3 (*p* = 0.031) compared to T0, while insomnia and fatigue showed significant improvements at T3, with *p*-values of 0.015 and 0.011, respectively (Table 3).

In the CG, the within-group analyses revealed some statistically significant variations only starting from T2 compared to baseline (T0). Specifically, changes were observed in role function (*p* = 0.043 at T2 and *p* = 0.028 at T3), emotional function at T2 (*p* = 0.041), social function at T3 (*p* = 0.017), and fatigue, which showed improvement at T2 (*p* = 0.046) (Table 4).

### 4.2. Secondary Outcomes

For the secondary outcomes, no statistically significant differences were observed between IG and CG at T0, indicating homogeneity between the two groups at the start of the study (Table 5 and Table 6).

The between-group analysis revealed statistically significant differences favoring the IG over the CG for the Six-Minute Walk Test (6MWT) and the Short Physical Performance Battery (SPPB) across the different follow-up time points. Specifically, for the 6MWT, significant differences were observed at T1 (*p* = 0.002), T2 (*p* = 0.012), and T3 (*p* = 0.003). Similarly, the SPPB showed significant differences at T1 (*p* = 0.003), T2 (*p* = 0.006), and T3 (*p* = 0.006) (Table 5). In contrast, no statistically significant differences were found between the two groups in anxiety, depression, or sleep quality, as measured by the Hospital Anxiety and Depression Scale (HADS) and the Pittsburgh Sleep Quality Index (PSQI), respectively (Table 5), nor in the 26 nutritional and body composition parameters analyzed via bioimpedance (Table 6).

The within-group analysis for the IG showed statistically significant improvements compared to baseline (T0) in the following parameters measured at different follow-up time points. The 6MWT demonstrated significant differences at T1 (*p* = 0.021) and T3 (*p* = 0.021), while the SPPB showed significant variations at T1 (*p* = 0.007), T2 (*p* = 0.034), and T3 (*p* = 0.018) compared to baseline. Regarding psychological parameters, anxiety levels, as measured by the HADS scale, were significantly reduced at T1 (*p* = 0.028) and T3 (*p* = 0.015), while a significant reduction in depression was observed at T3 (*p* = 0.017). Additionally, sleep quality (PSQI) showed a significant improvement at T1 (*p* = 0.006) compared to baseline (Table 7).

Regarding bioimpedance indices, both the phase angle and the phase angle standardized for sex and age showed significant improvements in IG at all follow-up time points compared to baseline: T1 (*p* = 0.005; *p* = 0.003), T2 (*p* = 0.008; *p* = 0.008), and T3 (*p* = 0.005; *p* = 0.005). Furthermore, the analysis revealed a significant reduction in the percentage of extracellular water (ECWpct) and an increase in the percentage of intracellular water (ICWpct) in IG at T1 (*p* = 0.003), T2 (*p* = 0.008), and T3 (*p* = 0.005). These changes were accompanied by an improvement in body cell mass (BCM) and body cell mass index (BCMI) at T1 (*p* = 0.013; *p* = 0.008), T2 (*p* = 0.011; *p* = 0.012), and T3 (*p* = 0.013; *p* = 0.005), compared to baseline. In this context, the sodium-to-potassium ratio (NaK) in IG showed a significant reduction at T1 (*p* = 0.011) and T3 (*p* = 0.010), suggesting an improvement in electrolyte balance (Table 8).

For the fat-free mass index (FFMI), a significant improvement was observed in IG at all follow-up time points, T1 (*p* = 0.023), T2 (*p* = 0.017), and T3 (*p* = 0.016), compared to baseline. This was accompanied by a significant increase in basal metabolic rate at T1 (*p* = 0.013), T2 (*p* = 0.013), and T3 (*p* = 0.011). Additionally, a significant variation in appendicular skeletal muscle mass (ASMM) was observed in IG at T3 (*p* = 0.028).

Finally, significant improvements were noted in IG for nutritional status at T1 (*p* = 0.010), T2 (*p* = 0.011), and T3 (*p* = 0.007), as well as in hydration status at T1 (*p* = 0.016), compared to baseline (Table 8).

Within-group analysis for CG revealed statistically significant improvements compared to baseline (T0) in a limited number of parameters. Specifically, for the anxiety subscale of the Hospital Anxiety and Depression Scale (HADS), a significant improvement was observed at T2 (*p* = 0.044), while for the Pittsburgh Sleep Quality Index (PSQI), significant differences were found at T2 (*p* = 0.027) and T3 (*p* = 0.026) (Table 9). No statistically significant differences were observed in bioimpedance parameters in CG compared to baseline (Table 10).

### 4.3. Feasibility and Safety of the Study

The study demonstrated good feasibility, with an eligibility rate of 52% (56 eligible patients out of 107 assessed) and a recruitment rate of 44.6%, as 25 out of 56 eligible patients agreed to participate. Notably, 31 patients declined participation for various reasons, including family-related motivations (22.6%), the perception of excessive time and energy commitment (16.1%), psychological state (12.9%), work commitments (12.9%), preference for independent and non-structured physical activity (9.7%), and other unspecified reasons (25.8%). The adherence rate to the exercise protocol for IG was 98% (305 sessions completed out of 312 scheduled sessions), and no adverse events were reported during the sessions. All patients assigned to IG completed the full evaluation protocol (T1, T2, T3) without any interruptions.

### 4.4. Postoperative Complications

No postoperative complications were observed in either group during follow-up, according to the Dindo-Clavien classification.

## 5. Discussion

The results of this study suggest that a combined and supervised home-based exercise program, initiated in the immediate post-surgical phase, has had a significant and positive impact on multiple dimensions of quality of life, functional capacity, and physical performance in patients with colon-rectal neoplasia undergoing laparoscopic resective surgery. These beneficial effects are consistent with existing scientific literature, which highlights how structured and supervised exercise programs can facilitate recovery and contribute to the improvement of well-being in oncological patients. Numerous studies have demonstrated that physical exercise can alleviate the side effects associated with anti-cancer treatments, significantly improving cardiorespiratory fitness, muscle strength, and, consequently, quality of life [42,43,44,45,46]. However, it is important to note that most research focuses on exercise programs administered during and/or after adjuvant treatment, usually weeks or months after surgical intervention [13]. In this context, studies investigating the role of rehabilitation in the immediate postoperative period remain limited, and the optimal modalities of physical exercise need further clarification [47]. Early postoperative mobilization is strongly recommended by Enhanced Recovery After Surgery (ERAS) guidelines [12], but rehabilitation programs are not currently part of the standard practice after surgical treatment for colon-rectal neoplasia.

Patients undergoing colon-rectal surgery often experience a reduction in physiological and functional capacity, even in the absence of complications, which negatively impacts quality of life (QoL) [48]. A systematic review has shown that QoL tends to decrease up to six months post-treatment, returning to baseline levels within a year [49]. It is hypothesized that the decreases in QoL in the first six months are attributable to post-treatment effects, which can persist for several months and include symptoms such as fatigue, appetite loss, and anxiety [50].

In the present study, a combined and supervised home-based exercise program lasting two months significantly improved both quality of life and role functioning in patients who had undergone colon-rectal cancer surgery, compared to the CG. Role functioning reflects an individual’s ability to engage in social and professional roles despite the limitations imposed by the disease and treatments. In oncology, improvements indicate a return to active participation in daily life, with fewer compromises in work and relational domains. A recent Cochrane review on the impact of physical activity in patients with non-advanced colon-rectal cancer (classified as T1-4 N0-2 M0) highlighted positive effects of physical exercise on aerobic fitness, fatigue, and quality of life in the immediate and short-term follow-up [13]. In our study, exploratory non-parametric analyses showed significant improvements in several quality-of-life domains at the end of the exercise program (T1) and two months after its conclusion (T2) in the IG compared to the CG. Specifically, at T1, significant improvements were found in role functioning, cognitive functioning, and fatigue management, as assessed by the EORTC QLQ-C30 scale. At T2, an additional improvement in physical functioning was observed, again favoring the IG. Intragroup analysis within the IG revealed that the most significant improvements occurred in the first four months after the exercise program began, particularly in areas related to quality of life, role functioning, physical, social, and emotional functioning, fatigue, and pain, while other symptomatic scales showed little variation over time (nausea/vomiting, constipation, diarrhea, and financial difficulties). These results are in line with observations from other studies, which documented a similar evolution in functional quality of life scales, highlighting the predominant improvement in role functioning [51].

In our study, the IG showed a significant reduction in fatigue in the early postoperative phase, at two months from the start of exercise, and a significant improvement in physical functioning at four months, compared to standard care. Fatigue is a common symptom, present in 60–96% of oncological patients during and after chemotherapy, radiotherapy, or surgery, and its impact on colorectal cancer (CRC) patients is particularly relevant [52,53]. It negatively affects mood and quality of life, interfering with daily activities [54]. Literature indicates that perceived work ability is inversely correlated with levels of fatigue in cancer survivors [55]. Our results support the evidence that supervised exercise improves fatigue compared to conventional therapies [56]. However, it is important to note that an improvement in fatigue was also recorded in the CG at four months from baseline, despite the absence of a rehabilitation intervention. This observation is consistent with a recent study showing that levels of fatigue in oncological patients can decrease spontaneously after anti-cancer treatment, even without specific interventions, likely due to the gradual reduction in inflammation associated with treatment [57]. Therefore, the results of this study suggest that the early integration of a physical exercise program could play a role in accelerating the functional recovery of CRC patients. While this observation requires further confirmation through prospective, randomized studies, it suggests that supervised exercise not only helps mitigate fatigue levels but may also facilitate a faster and more complete recovery of physical abilities, thus improving autonomy and quality of life for oncological patients. It is evident that some patients are more predisposed to develop cancer-related fatigue; several risk factors have already been identified, including advanced disease stage, female gender, younger age, type of surgery, and chemotherapy [58,59]. These factors should be considered during rehabilitation to identify patients who may need closer monitoring.

Furthermore, the results of our study showed a statistically significant improvement in the 6MWT in the IG compared to the CG at two, four-, and six-months post-baseline. The 6MWT is considered a modifiable risk factor for better postoperative recovery in patients undergoing colorectal surgery, demonstrating its predictive value in the clinical context [60,61,62,63,64]. Functional decline following surgery is one of the most recognized adverse effects in medicine [65] and is strongly related to the systemic inflammation induced by the surgical stress response [66]. Previous studies have shown that prehabilitation programs, including both aerobic and resistance exercise, have resulted in significant improvements in functional capacity before surgery, helping to reduce the risk of postoperative complications [17,67,68,69,70,71]. This suggests that targeted physical interventions can have positive effects both in the preoperative and postoperative periods. In line with this evidence, our data highlight how the early initiation of a supervised physical exercise program in the immediate postoperative period can improve functional capacity and facilitate postoperative recovery in CRC patients.

In addition to benefits on functional capacity, a significant improvement in the Short Physical Performance Battery (SPPB) was observed in the IG compared to standard care at all time points. The SPPB, which assesses balance, mobility, and strength, is an indicator of physical performance and has been shown to correlate significantly with survival in oncological patients, as highlighted in a systematic review [72]. Specifically, a 1-point improvement in total SPPB score is associated with a 12% relative reduction in mortality risk in cancer survivors [73]. Moreover, a recent prospective study shows that a decrease in physical performance, measured by the SPPB or handgrip strength, is frequently observed in colorectal cancer patients before surgery and is correlated with an increased risk of postoperative complications. These correlations suggest that physical performance tests could serve as prognostic tools, particularly in elderly patients, who often present a state of frailty [74].

From the analysis of bioimpedance data, no statistically significant differences were found between the groups analyzed. However, within the IG, significant variations were observed, suggesting a positive effect of the rehabilitation program on body composition and cellular functionality. Among the parameters analyzed, phase angle (PhA) and sex- and age-adjusted phase angle showed significant improvements at various assessment time points, compared to baseline, indicating an enhancement of cell membrane integrity and body fluid distribution [75]. In healthy populations, PhA typically ranges from 5 to 7 but tends to decrease with age due to muscle mass loss and changes in body fluid proportions [76]. Studies have demonstrated that lower PhA values are correlated with increased mortality and morbidity in various patient groups, highlighting its prognostic value in neoplastic and chronic diseases, including colorectal cancer (CRC) and prostate cancer (PC) [37,77,78].

The bioimpedance data analysis in the IG revealed significant changes in body composition, particularly a reduction in extracellular water percentage (ECWpct) and an increase in intracellular water percentage (ICWpct), associated with an improvement in body cell mass (BCM) and body cell mass index (BCMI) at T1, T2, and T3. These results can be interpreted positively, as a high ECW content has been associated with an unfavorable prognosis in cancer patients [79]. Moreover, recent studies have shown that a preoperative high ECW/total body water (TBW) ratio is a predictive factor for recurrence and poor survival in CRC, regardless of TNM stage [80]. Similarly, a low ICW/TBW ratio, indicative of cellular dehydration and increased extracellular osmotic pressure, has been linked to higher all-cause mortality [81]. In our study, the significant increase in ICW in the IG, observed at all follow-up times, suggests an improvement in cellular functionality and protein synthesis, indicative of tissue recovery and improved intracellular fluid balance [82,83]. The reduction in ECW may reflect a decrease in edema and inflammation, conditions that have been correlated with poor prognosis in oncology patients [80]. Additionally, the increase in BCM and BCMI highlights an increase in metabolically active mass, essential for muscle function and postoperative recovery.

Regarding fat-free mass index (FFMI), significant improvement was recorded in the IG at all follow-up time points, compared to baseline. However, it is important to note that the assessment of fat-free mass by bioimpedance (BIA) is debated. While one study showed good concordance between fat-free mass estimated by BIA and DXA, provided appropriate equations are used [84], a subsequent study by Bärebring et al. highlighted limitations in BIA accuracy for quantifying fat-free mass changes in non-metastatic CRC patients [85]. Therefore, despite the improvement in FFMI observed in our study suggesting an increase in fat-free mass, it is important to consider the variability of measurement methods used to ensure the accuracy of the results.

Finally, the significant increase in appendicular skeletal muscle mass (ASMM) at T3 in the IG, compared to baseline, holds further clinical relevance, as ASMM represents over 75% of total skeletal muscle mass and plays a crucial role in maintaining physical capacity and motor function. An increase in ASMM is correlated with improvements in mobility and the ability to perform daily activities, contributing to a better quality of life in oncology patients [86]. In contrast, low ASMM levels are associated with frailty, disability, and reduced bone mineral density, increasing the risk of osteoporosis and fractures [87]. These data suggest that the implementation of targeted rehabilitation interventions may have positive effects on muscle mass, with favorable impacts on overall patient health.

The feasibility of rehabilitation programs in the surgical population, especially among the elderly with muscle weakness and physical inactivity, is frequently a concern in the scientific community. These concerns are amplified by the presence of nutritional, cardiovascular, and metabolic comorbidities, often found in CRC patients, which may limit their active participation in physical exercise programs. However, recent evidence, including data from the POLARIS study (Predicting Optimal Cancer Rehabilitation and Supportive Care), suggests that patients with the lowest physical functionality experience the greatest benefits from physical exercise, demonstrating that a targeted rehabilitation approach can yield positive outcomes even in elderly individuals with physical limitations [88,89].

Our study showed good feasibility, with an eligibility rate of 52% and a recruitment rate of 44.6%. These results align with previous studies on physical activity in colorectal cancer (37–41%) [24,90] and prostate cancer (37%) [91] and are higher than a breast cancer study (19%) [92]. We observed excellent adherence to the home-based supervised exercise program (98%). In the literature, an adherence rate ≥ 80% is considered high, according to previous studies on oncology patients [70,93,94]. The enrolled patients showed high motivation to participate in the sessions. No adverse effects were observed in the IG during the rehabilitation period.

This study followed the recommendations of the American College of Sports Medicine [20] and the Canadian Society for Exercise Physiology [21] regarding exercise type, intensity, and duration. A supervised exercise mode was adopted, as it has been shown in the literature to achieve superior outcomes compared to unsupervised exercise [95]. The decision to employ a distance intervention via a home-based exercise program, supervised through telemedicine, was driven by the need to mitigate logistical barriers, such as geographical distance, which previous feasibility studies have frequently identified as a key factor contributing to reduced participation rates [96,97]. In a previous study conducted by our research group, a supervised exercise program was performed in person at a dedicated facility [98]. Despite following a similar approach regarding exercise type, intensity, and duration, the recruitment rate was significantly lower at 29%, with only 13 patients enrolled out of 38 eligible, primarily due to practical challenges such as geographical distance, reported by 66.7% of patients as a barrier [98]. These findings emphasize the limitations of in-person interventions and underscore the potential of telemedicine-based approaches to overcome logistical barriers, enhancing both accessibility and participation.

Moreover, the implementation of a home-based supervised exercise program for colorectal cancer patients in the early postoperative phase may offer significant cost-effectiveness compared to traditional rehabilitation models. Traditional rehabilitation often requires patients to attend outpatient clinics or specialized rehabilitation centers, incurring costs related to travel, clinic visits, and facility use. In contrast, a home-based exercise program can reduce these logistical and financial burdens, making it more accessible for patients, especially those in remote areas or with limited mobility. Additionally, home-based exercise programs are typically less resource-intensive, requiring minimal equipment, and can be tailored to individual patient needs. This flexibility not only lowers the cost of delivery but also improves patient adherence, potentially leading to better health outcomes and reducing the need for more expensive medical interventions.

This study has several limitations, including the small sample size, the lack of randomization, and the absence of a blinded evaluator. Additionally, some participants received adjuvant chemotherapy during the study period (69.2% in the IG and 50% in the CG), but no stratified analysis was conducted to assess whether and how this treatment influenced the effectiveness of the intervention. Although the preliminary results should be interpreted with caution, they suggest a positive effect of supervised home-based exercise in the early postoperative phase in colorectal cancer patients. The data obtained from the descriptive and exploratory analyses encourage us to plan a randomized controlled trial to confirm these results and evaluate the effectiveness of the intervention. Future studies could also include preoperative assessments to further investigate the relationship between surgery, functional decline, and quality of life. A larger sample, participant randomization, the use of blinded evaluators, and the analysis of long-term effects, as well as stratified analyses based on adjuvant treatments, would be important steps to improve the validity of the results and the understanding of the sustained effectiveness of the intervention.

## 6. Conclusions

Peri-rehabilitation is an emerging field in cancer rehabilitation, involving a process of pre- and post-surgical optimization, aimed at improving functional capacity and tolerance to stressful events such as surgery and subsequent anti-cancer treatments. An early postoperative rehabilitation phase could be an effective tool to help patients with colon cancer undergoing laparoscopic resective surgery improve functional recovery, reduce fatigue, and enhance overall quality of life, optimally preparing them for the management of further anti-cancer treatments.

## Figures and Tables

**Table 1:** Demographic and clinical characteristics of colorectal cancer patients at baseline.

Variables	Intervention Group (IG) *n* = 13	Control Group (CG) *n* = 10	*p*-Value
Age (years)—median (range)	65.0 (56.0–80.0)	74.5 (71.0–78.0)	0.580
Gender—*n*. (%)			
Male	8 (61.5)	6 (60.0)	
Female	5 (38.5)	4 (40.0)	
BMI—median (range)	22.7 (20.1–25.7)	26.25 (24.2–28.3)	0.151
Type of neoplasm—*n*. (%)			
Colon	10 (76.9)	7 (70.0)	
Rectum	3 (23.1)	3 (30.0)	
TNM staging—*n*. (%)			
IIA	2 (15.4)	2 (20.0)	
IIB	4 (30.8)	2 (20.0)	
IIIB	7 (53.8)	6 (60.0)	
Type of resection—*n*. (%)			
Colon	10 (76.9)	7 (70.0)	
Rectum	3 (23.1)	3 (30.0)	
New stoma—*n*. (%)	0 (0.0)	0 (0.0)	
Chemotherapy—*n*. (%)	9 (69.2)	5 (50.0)	

BMI, Body Mass Index; TNM, Tumor, Node, Metastasis.

**Table 2:** EORTC QLQ-C30: analysis between IG and GC at baseline (T0), 2 months (T1), 4 months (T2), and 6 months (T3) from baseline. Significance is established at *p* < 0.05 (bold numbers).

Variables	*p*-Value			
	Between Analysis			
	T0	T1	T2	T3
Quality of life	0.169	0.155	0.291	0.686
Physical functioning	0.732	0.068	**0.006**	0.558
Role functioning	0.574	**0.007**	0.129	0.260
Emotional functioning	0.365	0.410	0.678	0.751
Cognitive functioning	0.116	**0.013**	0.882	0.822
Social functioning	0.156	0.324	0.295	0.410
Fatigue	0.998	**0.004**	0.770	0.529
Nausea and vomiting	0.380	0.492	0.350	0.399
Pain	0.633	0.122	0.299	0.672
Dyspnea	0.748	0.525	0.171	0.143
Insomnia	0.917	0.567	0.507	0.265
Appetite loss	0.690	0.561	0.171	0.713
Constipation	0.454	0.142	0.507	0.998
Diarrhea	0.823	0.782	0.562	0.412
Financial difficulties	0.862	0.258	0.507	0.338

**Table 3:** EORTC QLQ-C30: within IG analysis 2 months after the start of the physical exercise program (T1) and 2 (T2) and 4 (T3) months after the end of the physical exercise program compared to baseline (T0). Data are presented as median and range. * Increasing values correspond to an improvement, ** Increasing values correspond to a worsening. Significance is established at *p* < 0.05 (bold numbers).

Variables (Median, Range)	Intervention Group (IG)				*p*-Value		
					Within IG		
	T0	T1	T2	T3	T0–T1	T0–T2	T0–T3
* Quality of life	66.7	70.85	83.3	83.3	**0.014**	**0.018**	**0.014**
	(33.3–83.3)	(58.3–100.0)	(67.0–92.0)	(67.0–100.0)			
* Physical functioning	93	90.15	96.6	93.3	**0.021**	**0.018**	0.161
	(60.0–100.0)	(80.0–100.0)	(87.0–100.0)	(87.0–100.0)			
* Role functioning	75.0	100.0	100.0	100.0	**0.007**	**0.043**	**0.012**
	(34.0–100.0)	(83.3–100.0)	(83.3–100.0)	(100.0–100.0)			
* Emotional functioning	87.8	95.8	87.5	91.7	**0.041**	0.173	**0.046**
	(42.0–93.0)	(75.0–100.0)	(67.0–100.0)	(83.0–100.0)			
* Cognitive functioning	100.0	100.0	100.0	100.0	0.498	1.000	0.715
	(83.0–100.0)	(83.0–100.0)	(83.0–100.0)	(83.0–100.0)			
* Social functioning	75.0	100.0	100.0	100.0	0.065	**0.026**	**0.011**
	(50.0–100.0)	(66.7–100.0)	(83.0–100.0)	(100.0–100.0)			
** Fatigue	33.3	22.2	11.0	11.1	0.054	0.080	**0.011**
	(0.0–44.4)	(0.0–33.3)	(0.0–33.0)	(0.0–33.0)			
** Nausea and vomiting	0.0	0.0	0.0	0.0	0.102	0.180	0.465
	(0.0–33.3)	(0.0–16.6)	(0.0–0.0)	(0.0–17.0)			
** Pain	16.7	0.0	0.0	0.0	**0.030**	0.334	**0.031**
	(0.0–33.3)	(0.0–16.7)	(0.0–33.3)	(0.0–17.0)			
** Dyspnea	0.0	0.0	0.0	0.0	0.102	0.180	0.157
	(0.0–33.3)	(0.0–33.3)	(0.0–0.0)	(0.0–0.0)			
** Insomnia	53.3	0.0	0.0	0.0	0.170	0.059	**0.015**
	(0.0–66.7)	(0.0–33.3)	(0.0–33.3)	(0.0–33.3)			
** Appetite loss	0.0	0.0	0.0	0.0	0.072	0.102	0.121
	(0.0–66.7)	(0.0–33.3)	(0.0–0.0)	(0.0–33.0)			
** Constipation	0.0	0.0	0.0	0.0	0.096	0.317	0.131
	(0.0–33.3)	(0.0–0.0)	(0.0–33.3)	(0.0–33.3)			
** Diarrhea	33.3	16.6	0.0	0.0	0.655	0.180	0.480
	(0.0–33.3)	(0.0–33.3)	(0.0–33.3)	(0.0–33.3)			
** Financial Difficulties	0.0	0.0	0.0	0.0	0.157	0.998	0.317
	(0.0–33.3)	(0.0–33.3)	(0.0–33.3)	(0.0–33.3)			

**Table 4:** EORTC QLQ-C30: within analysis in the CG at 2 months (T1), 4 months (T2), and 6 months (T3) from baseline (T0). Data are presented as median and range. * Increasing values correspond to an improvement, ** Increasing values correspond to a worsening.

Variables (Median, Range)	Control Group (CG)				*p*-Value		
					Within CG		
	T0	T1	T2	T3	T0–T1	T0–T2	T0–T3
* Quality of life	66.7	58.3	66.7	75.0	0.345	0.500	0.223
	(33.3–91.7)	(33.3–91.7)	(17.0–92.0)	(33.0–100.0)			
* Physical functioning	87	80.0	73.3	87.0	0.059	0.225	0.465
	(53.3–93.3)	(53.33–87.0)	(53.0–87.0)	(53.0–100.0)			
* Role functioning	50.0	83.0	100.0	100.0	0.093	0.043	0.028
	(0.0–100.0)	(33.3–84.0)	(33.3–100.0)	(33.0–100.0)			
* Emotional functioning	66.7	75.0	91.7	87.0	0.713	0.041	0.345
	(41.7–83.3)	(41.7–91.7)	(42.0–100.0)	(42.0–100.0)			
* Cognitive functioning	83.3	83.0	100.0	100.0	0.276	0.066	0.066
	(66.7–100.0)	(66.6–100.0)	(67.0–100.0)	(67.0–100.0)			
* Social functioning	100.0	66.7	85.0	100.0	0.588	0.500	0.017
	(33.3–100.0)	(50.0–100.0)	(67.0–100.0)	(66.7–100.0)			
** Fatigue	44.4	44.4	22.2	33.0	0.285	0.046	0.080
	(22.0–77.8)	(33.3–77.8)	(11.0–78.0)	(0.0–78.0)			
** Nausea and vomiting	0.0	0.0	0.0	0.0	0.998	0.317	0.785
	(0.0–33.3)	(0.0–16.6)	(0.0–33.3)	(0.0–33.0)			
** Pain	33.3	16.7	0.0	0.0	0.273	0.074	0.066
	(0.0–66.7)	(0.0–50.0)	(0.0–50.0)	(0.0–17.0)			
** Dyspnea	0.0	0.0	0.0	0.0	0.180	0.083	0.317
	(0.0–100.0)	(0.0–33.3)	(0.0–66.7)	(0.0–67.0)			
** Insomnia	33.3	33.3	0.0	33.3	0.998	0.246	0.998
	(0.0–66.7)	(0.0–66.7)	(0.0–33.3)	(0.0–67.0)			
** Appetite loss	0.0	0.0	0.0	0.0	0.371	0.705	0.157
	(0.0–100.0)	(0.0–100.0)	(0.0–100.0)	(0.0–100.0)			
** Constipation	0.0	0.0	0.0	0.0	0.655	0.593	0.998
	(0.0–66.7)	(0.0–0.0)	(0.0–33.3)	(0.0–33.3)			
** Diarrhea	0.0	0.0	0.0	0.0	0.317	0.655	0.317
	(0.0–0.0)	(0.0–33.3)	(0.0–33.3)	(0.0–66.6)			
** Financial difficulties	0.0	0.0	0.0	0.0	0.317	0.157	0.285
	(0.0–33.3)	(0.0–66.7)	(0.0–33.0)	(0.0–33.0)			

**Table 5:** Functional and psychological outcomes: analysis between IG and CG at baseline (T0), 2 months (T1), 4 months (T2), and 6 months (T3) from baseline.

Variables	*p*-Value			
	Between Analysis			
	T0	T1	T2	T3
6MWT (m)	0.078	**0.002**	**0.012**	**0.003**
SPPB	0.752	**0.003**	**0.006**	**0.006**
Handgrip Strength Test (kg)	0.901	0.885	0.309	0.512
HADS Anxiety scale	0.596	0.215	0.640	0.370
HADS Depression scale	0.950	0.636	0.380	0.528
PSQI	0.332	0.440	0.254	0.752

Abbreviations: 6MWT, 6-Minute Walking Test; SPPB, Short Physical Performance Battery; SMI, skeletal muscle mass index; BIA, bioimpedance analysis; PhA, phase angle; HADS, Hospital Anxiety and Depression Scale; PSQI, Pittsburgh Sleep Quality Index. Significance is established at *p* < 0.05 (bold numbers).

**Table 6:** Bioimpedance outcomes: between-group analysis in the IG and CG at baseline (T0), 2 months (T1), 4 months (T2), and 6 months (T3) from baseline.

Variables	*p*-Value			
	Between Analysis			
	T0	T1	T2	T3
Rz	0.616	0.242	0.280	0.258
Xc	0.973	0.938	0.817	0.671
FFM	0.483	0.102	0.537	0.480
TBW	0.570	0.119	0.537	0.479
ECW	0.462	0.310	0.998	0.887
BCM	0.973	0.243	0.440	0.396
FM	0.285	0.484	0.487	0.777
PA	0.867	0.309	0.089	0.670
NaK	0.370	0.936	0.449	0.821
FMpct	0.151	0.585	0.216	0.203
FFMpct	0.151	0.585	0.216	0.203
TBWpct	0.171	0.755	0.280	0.321
ECWpct	0.894	0.309	0.089	0.777
ICWpct	0.894	0.309	0.089	0.777
MM	0.920	0.243	0.440	0.357
MMpct	0.133	0.640	0.643	0.888
BM	0.963	0.243	0.440	0.396
BCMI	0.593	0.160	0.188	0.179
Hydration	0.737	0.814	0.436	0.998
Nutrition	0.815	0.186	0.355	0.322
SMI	0.688	0.185	0.247	0.228
SMM	0.920	0.243	0.440	0.357
ASMM	0.504	0.185	0.537	0.479
FMI	0.191	0.533	0.757	0.776
FFMI	0.332	0.139	0.279	0.179
SPA	0.896	0.938	0.246	0.777

Abbreviations: Rz, Resistance; Xc, Reactance; FFM, Fat-Free Mass; TBW, Total Body Water; ECW, Extracellular Water; BCM, Body Cell Mass; FM, Fat Mass; PA, Phase Angle; NaK, Sodium/Potassium Ratio; FMpct, Fat Mass Percentage; FFMpct, Fat-Free Mass Percentage; TBWpct, Total Body Water Percentage; ECWpct, Extracellular Water Percentage; ICWpct, Intracellular Water Percentage; MM, Muscle Mass; MMpct, Muscle Mass Percentage; BM, Basal Metabolism; BCMI, Body Cell Mass Index; Hydration, Hydration Status; Nutrition, Nutritional Status; SMI, Skeletal Muscle Mass Index; SMM, Skeletal Muscle Mass; ASMM, Appendicular Skeletal Muscle Mass; FMI, Fat Mass Index; FFMI, Fat-Free Mass Index; SPA, Standardized Phase Angle. Statistical significance is set at *p* < 0.05.

**Table 7:** Functional and psychological outcomes: within-group analysis in the intervention group (IG) at 2 months from the start of the exercise program (T1) and at 2 (T2) and 4 (T3) months after the end of the exercise program compared to baseline (T0).

Variables (Median, Range)	Intervention Group (IG)				*p*-Value		
					Within IG		
	T0	T1	T2	T3	T0–T1	T0–T2	T0–T3
6MWT (m)	490.0	590	572.5	580	**0.021**	0.091	**0.021**
	(360–685)	(360–815)	(445–815)	(445–750)			
SPPB	10.5	11.00	11.5	12.0	**0.007**	**0.034**	**0.018**
	(7.0–12.0)	(10.0–12.0)	(10.0–12.0)	(10.0–12.0)			
Handgrip Strength Test (kg)	57.3	57.05	56.65	55.5	0.059	0.374	0.333
	(28.3–61.7)	(28.3–61.5)	(37–62.0)	(38.6–65.3)			
HADS—Anxiety	4.5	5.0	4.0	3.0	**0.028**	0.414	**0.015**
	(3.0–9.0)	(0.0–8.0)	(2.0–9.0)	(1.0–5.0)			
HADS—Depression	3.0	3.5	1.5	2.0	0.697	0.673	**0.017**
	(2.0–9.0)	(1.0–10.0)	(0.0–16.0)	(1.0–3.0)			
PSQI	7.0	5.0	3.0	4.0	**0.006**	0.058	0.084
	(2.0–11.0)	(3.0–7.0)	(1.0–7.0)	(3.0–6.0)			

Abbreviations: 6MWT, 6-Minute Walking Test; SPPB, Short Physical Performance Battery; SMI, Skeletal Muscle Mass Index; BIA, Bioelectrical Impedance Analysis; PhA, Phase Angle; HADS, Hospital Anxiety and Depression Scale; PSQI, Pittsburgh Sleep Quality Index. Data are presented as median and range. Statistical significance is set at *p* < 0.05 (bold numbers).

**Table 8:** Bioimpedance outcomes: within-group analysis in the intervention group (IG) at 2 months from the start of the exercise program (T1) and at 2 (T2) and 4 (T3) months after the end of the exercise program compared to baseline (T0).

Variables (Median, Range)	Intervention Group (IG)				*p*-Value		
					Within IG		
	T0	T1	T2	T3	T0–T1	T0–T2	T0–T3
Rz	523.0	506.2	486.6	507.7	0.062	0.066	0.203
	(478.1–583)	(436.8–557.0)	(453.0–557.0)	(493.8–561)			
Xc	49.6	51.25	49.5	53.6	**0.013**	0.326	0.173
	(41.0–56.3)	(44.0–60.2)	(44.0–59.3)	(49.1–59.4)			
FFM	54.3	53.8	52.7	54.0	0.091	0.066	0.059
	(46.1–57.2)	(48.5–61.0)	(48.2–61.0)	(47.2–58.2)			
TBW	39.9	39.6	38.9	39.6	0.266	0.097	0.109
	(33.9–42.0)	(35.5–44.8)	(35.3–44.8)	(34.6–42.7)			
ECW	19.3	18.2	18.5	18.4	**0.011**	0.343	0.236
	(16.3–22.5)	(15.6–20.5)	(15.7–20.7)	(15.7–19.9)			
BCM	26.5	28.1	26.6	28.5	**0.013**	**0.011**	**0.013**
	(23.0–29.8)	(26.4–34.4)	(26.4–34.4)	(25.5–31.0)			
FM	14.5	8.8	9.7	13.5	0.050	0.314	0.441
	(11.0–16.9)	(7.3–19.8)	(7.3–17.9)	(10.8–22.6)			
PA	5.6	6.1	5.8	5.9	**0.005**	**0.008**	**0.005**
	(4.1–5.7)	(5.0–6.5)	(5.0–6.5)	(5.7–6.1)			
NaK	1.15	1.10	1.10	1.10	**0.011**	0.059	**0.010**
	(1.1–1.4)	(1.0–1.4)	(1.0–1.4)	(1.0–1.2)			
FMpct	20.8	14.4	16.5	20.2	0.068	0.314	0.646
	(18.3–23.7)	(11.8–26.1)	(11.8–24.5)	(16.3–28.2)			
FFMpct	79.2	85.5	83.5	79.7	0.068	0.314	0.646
	(76.3–81.7)	(73.9–88.2)	(75.5–88.2)	(71.8–83.7)			
TBWpct	58.1	62.8	61.4	58.5	0.168	0.374	0.575
	(56.3–60.3)	(54.2–65.0)	(55.1–65.0)	(52.6–61.5)			
ECWpct	47.7	44.85	46.3	46.0	**0.003**	**0.008**	**0.005**
	(47.2–56.3)	(43.3–50.9)	(43.3–50.9)	(45.0–47.2)			
ICWpct	52.3	55.1	53.6	53.9	**0.003**	**0.008**	**0.005**
	(43.7–52.8)	(49.1–56.7)	(49.1–56.7)	(52.8–55.0)			
MM	26.7	25.7	26.1	25.2	0.168	0.086	0.444
	(21.1–29.1)	(22.9–32.2)	(22.6–32.2)	(21.8–29.6)			
MMpct	37.8	41.1	39.4	37.5	0.197	0.260	**0.005**
	(36.3–40.9)	(33.8–46.5)	(36.1–46.5)	(32.3–42.6)			
BM	1519	1564	1521.9	1576.5	**0.013**	**0.013**	**0.011**
	(1417–1612)	(1514–1748)	(1514–1748)	(1488–1649)			
BCMI	9.2	10.0	9.7	9.9	**0.008**	**0.012**	**0.005**
	(7.4–10.5)	(8.7–11.2)	(8.7–11.2)	(8.8–10.7)			
Hydration	73.4	73.3	74.4	73.3	**0.016**	0.246	0.105
	(73.3–73.9)	(73.0–73.7)	(73.0–73.8)	(73.1–73.5)			
Nutrition	790.7	847.2	812.5	872.3	**0.010**	**0.011**	**0.007**
	(668.8–916)	(765–1020)	(750.0–1020)	(720.9–931)			
SMI	8.8	9.2	9.1	9.1	0.091	0.057	0.202
	(7.3–9.6)	(7.9–10.5)	(7.8–10.5)	(7.6–9.7)			
SMM	26.7	25.7	26.1	25.2	0.168	0.086	0.444
	(22.1–29.1)	(22.9–32.2)	(22.6–32.2)	(21.8–29.6)			
ASMM	19.65	19.5	19.1	19.9	0.091	0.051	**0.028**
	(17.1–22.8)	(18.5–24.7)	(18.1–24.7)	(17.6–23.2)			
FMI	4.9	3.0	3.4	4.6	0.075	0.477	0.308
	(4.0–5.4)	(2.4–7.0)	(2.4–6.3)	(3.7–8.0)			
FFMI	18.0	18.7	18.3	18.8	**0.023**	**0.017**	**0.016**
	(16.0–20.2)	(16.8–19.9)	(16.7–21.3)	(16.3–20.3)			
SPA	0.45	0.70	0.70	0.75	**0.003**	**0.008**	**0.005**
	(−2.8–1.8)	(−1.7–3.7)	(−1.7–2.6)	(−0.4–3.2)			

Abbreviations: Rz, Resistance; Xc, Reactance; FFM, Fat-Free Mass; TBW, Total Body Water; ECW, Extracellular Water; BCM, Body Cell Mass; FM, Fat Mass; PA, Phase Angle; NaK, Sodium/Potassium Ratio; FMpct, Fat Mass Percentage; FFMpct, Fat-Free Mass Percentage; TBWpct, Total Body Water Percentage; ECWpct, Extracellular Water Percentage; ICWpct, Intracellular Water Percentage; MM, Muscle Mass; MMpct, Muscle Mass Percentage; BM, Basal Metabolism; BCMI, Body Cell Mass Index; Hydration, Hydration Status; Nutrition, Nutritional Status; SMI, Skeletal Muscle Mass Index; SMM, Skeletal Muscle Mass; ASMM, Appendicular Skeletal Muscle Mass; FMI, Fat Mass Index; FFMI, Fat-Free Mass Index; SPA, Standardized Phase Angle. Statistical significance is set at *p* < 0.05 (bold numbers).

**Table 9:** Functional and psychological outcomes: within-group analysis in the control group (CG) at 2 months (T1), 4 months (T2), and 6 months (T3) from baseline compared to baseline (T0).

Variables (Median, Range)	Control Group (CG)				*p*-Value		
					Within CG		
	T0	T1	T2	T3	T0–T1	T0–T2	T0–T3
6MWT (m)	402.5	400.0	424.2	405	0.074	0.462	0.463
	(330–560)	(290–510)	(300–535)	(310–505)			
SPPB	10.5	10.00	9.5	10.0	0.480	0.301	0.394
	(8.0–11.0)	(9.0–11.0)	(9.0–12.0)	(7.0–12.0)			
Handgrip Strength Test (kg)	41.0	41.0	40.5	40.0	0.893	0.753	0.866
	(20.7–42.9)	(18.3–42.0)	(22.0–42.0)	(19.7–42.0)			
HADS—Anxiety	7.0	6.0	5.0	5.0	0.416	**0.044**	0.293
	(3.0–14.0)	(1.0–14.0)	(0.0–14.0)	(0.0–14.0)			
HADS—Depression	6.0	6.0	4.0	5.0	0.345	0.674	0.500
	(4.0–9.0)	(1.0–9.0)	(0.0–13.0)	(0.0–9.0)			
PSQI	7.0	5.0	4.0	5.0	0.461	**0.027**	**0.026**
	(3.0–14.0)	(4.0–12.0)	(2.0–8.0)	(2.0–9.0)			

Abbreviations: 6MWT, 6-Minute Walking Test; SPPB, Short Physical Performance Battery; SMI, Skeletal Muscle Mass Index; BIA, Bioimpedance Analysis; PhA, Phase Angle; HADS, Hospital Anxiety and Depression Scale; PSQI, Pittsburgh Sleep Quality Index. Data are presented as median and range. Statistical significance is set at *p* < 0.05 (bold numbers).

**Table 10:** Bioimpedance outcomes: within-group analysis in the control group (CG) at 2 months (T1), 4 months (T2), and 6 months (T3) from baseline compared to baseline (T0).

Variables (Median, Range)	Control Group (CG)				*p*-Value		
					Within CG		
	T0	T1	T2	T3	T0–T1	T0–T2	T0–T3
Rz	407.4	436.5	387.3	424.0	0.998	0.068	0.715
	(395.2–419.6)	(416.7–456.4)	(358.3–416.4)	(396.2–451)			
Xc	46.1	51.65	44.8	44.5	0.998	0.465	0.998
	(40.4–51.8)	(50.6–52.7)	(41.4–48.2)	(44.2–44.8)			
FFM	62.3	60.1	64.4	60.9	0.998	0.068	0.998
	(58.8–65.8)	(56.0–64.2)	(59.0–69.0)	(56.0–65.9)			
TBW	45.8	44.0	47.45	44.9	0.999	0.068	0.998
	(43.1–48.5)	(41.0–47.1)	(43.3–51.6)	(41.2–48.6)			
ECW	20.1	18.7	20.4	20.4	0.999	0.705	0.715
	(17.8–22.5)	(17.7–19.7)	(18.6–22.2)	(19.5–21.4)			
BCM	34.6	34.5	36.4	32.8	0.998	0.144	0.715
	(34.6–34.8)	(31.7–37.3)	(33.4–39.5)	(29.1–36.6)			
FM	10.7	13.1	8.8	12.3	0.998	0.068	0.273
	(7.2–14.2)	(10.0–16.3)	(7.0–10.6)	(10.0–14.6)			
PA	6.4	6.7	6.6	6.0	0.999	0.273	0.715
	(5.8–7.0)	(6.6–6.9)	(6.6–6.6)	(5.7–6.4)			
NaK	1.15	1.05	1.10	1.15	0.655	0.998	0.998
	(1.1–1.2)	(1.0–1.1)	(1.1–1.1)	(1.1–1.2)			
FMpct	14.35	17.7	11.9	16.6	0.998	0.068	0.715
	(10.9–17.8)	(15.2–20.2)	(10.7–13.2)	(15.1–18.1)			
FFMpct	85.6	82.3	88.0	83.4	0.998	0.068	0.715
	(82.2–89.1)	(79.8–84.8)	(86.8–89.3)	(81.9–84.9)			
TBWpct	63.0	60.3	64.8	61.4	0.999	0.066	0.715
	(60.7–65.3)	(58.5–62.1)	(64.0–65.6)	(60.3–62.5)			
ECWpct	43.9	42.4	43.1	45.7	0.998	0.357	0.715
	(41.4–46.5)	(41.8–43.1)	(43.1–43.1)	(44.1–47.3)			
ICWpct	56.0	57.5	56.9	54.3	0.998	0.357	0.715
	(53.5–58.6)	(56.9–58.2)	(56.9–56.9)	(52.7–55.9)			
MM	31.0	29.2	32.5	30.0	0.998	0.068	0.581
	(29.9–32.1)	(27.8–30.6)	(30.1–35.0)	(28.0–32.0)			
MMpct	42.7	40.0	44.5	41.1	0.998	0.144	0.715
	(40.1–45.3)	(3.0–42.1)	(43.5–45.6)	(39.8–42.5)			
BM	1753.9	1749.7	1807.55	1703.6	0.998	0.144	0.715
	(1748–1759)	(1668–1831)	(1718–1896)	(1595–1811)			
BCMI	12.45	12.4	13.1	11.8	0.998	0.141	0.715
	(12.3–12.6)	(11.6–13.2)	(12.3–14.0)	(10.7–13.0)			
Hydration	73.5	73.2	73.6	73.6	0.655	0.276	0.705
	(73.2–73.8)	(73.2–73.3)	(73.4–73.8)	(73.6–73.6)			
Nutrition	1079.1	1073.8	1135.9	1024.0	0.998	0.144	0.715
	(1075–1083)	(995.7–1152)	(1050–1221)	(916.9–1031)			
SMI	11.2	10.5	11.75	10.8	0.998	0.068	0.713
	(11.0–11.4)	(10.2–10.8)	(11.1–12.4)	(10.3–11.3)			
SMM	31.0	29.2	32.55	30.0	0.998	0.068	0.581
	(29.9–32.1)	(27.8–30.6)	(30.1–35.0)	(28.0–32.0)			
ASMM	22.7	22.1	23.6	22.1	0.998	0.144	0.715
	(21.7–23.8)	(20.6–23.7)	(21.6–25.6)	(20.2–24.1)			
FMI	3.8	4.75	3.2	4.4	0.998	0.180	0.269
	(2.6–5.0)	(3.7–5.8)	(2.6–3.8)	(3.7–5.2)			
FFMI	22.4	21.7	23.25	22.0	0.998	0.068	0.998
	(21.6–23.3)	(20.6–22.8)	(21.7–24.8)	(20.6–23.4)			
SPA	1.4	1.8	1.6	0.9	0.998	0.197	0.715
	(0.6–2.2)	(1.7–2.0)	(1.5–1.7)	(0.6–1.3)			

Abbreviations: Rz, Resistance; Xc, Reactance; FFM, Fat-Free Mass; TBW, Total Body Water; ECW, Extracellular Water; BCM, Body Cell Mass; FM, Fat Mass; PA, Phase Angle; NaK, Sodium/Potassium Ratio; FMpct, Fat Mass Percentage; FFMpct, Fat-Free Mass Percentage; TBWpct, Total Body Water Percentage; ECWpct, Extracellular Water Percentage; ICWpct, Intracellular Water Percentage; MM, Muscle Mass; MMpct, Muscle Mass Percentage; BM, Basal Metabolism; BCMI, Body Cell Mass Index; Hydration, Hydration Status; Nutrition, Nutritional Status; SMI, Skeletal Muscle Mass Index; SMM, Skeletal Muscle Mass; ASMM, Appendicular Skeletal Muscle Mass; FMI, Fat Mass Index; FFMI, Fat-Free Mass Index; SPA, Standardized Phase Angle. Statistical significance is set at *p* < 0.05.

## Data Availability

The data presented in this study are available on request from the corresponding author.
